# Desmoplastic small round cell tumors: Multimodality treatment and new risk factors

**DOI:** 10.1002/cam4.1940

**Published:** 2019-01-16

**Authors:** Monika Scheer, Christian Vokuhl, Bernd Blank, Erika Hallmen, Thekla von Kalle, Marc Münter, Rüdiger Wessalowski, Maite Hartwig, Monika Sparber‐Sauer, Paul‐Gerhardt Schlegel, Christof M. Kramm, Udo Kontny, Bernd Spriewald, Thomas Kegel, Sebastian Bauer, Bernarda Kazanowska, Felix Niggli, Ruth Ladenstein, Gustaf Ljungman, Kirsi Jahnukainen, Jörg Fuchs, Stefan S. Bielack, Thomas Klingebiel, Ewa Koscielniak, the Cooperative Weichteilsarkom Studiengruppe [CWS]

**Affiliations:** ^1^ Pediatrics 5 Olgahospital Klinikum Stuttgart Stuttgart Germany; ^2^ Kiel Peadiatric Tumour Registry, Department of Pediatric Pathology University Hospital Kiel Germany; ^3^ Radiologisches Institut Olgahospital Klinikum Stuttgart Stuttgart Germany; ^4^ Radiation Oncology Klinikum Stuttgart Stuttgart Germany; ^5^ Pediatric Oncology Clinic Heinrich‐Heine‐University Düsseldorf Düsseldorf Germany; ^6^ Pediatric Hematology and Oncology University Hamburg‐Eppendorf Hamburg Germany; ^7^ Children's Hospital, Pediatric Oncology University Würzburg Würzburg Germany; ^8^ Pediatric Hematology and Oncology University Medical Center Göttingen Göttingen Germany; ^9^ Pediatric Hematology and Oncology University Medical Center Aachen Aachen Germany; ^10^ Internal Medicine 5 University Hospital Erlangen Erlangen Germany; ^11^ Hematology/Oncology University of Halle Halle Germany; ^12^ Sarcoma Center, West German Cancer Center University of Duisburg‐Essen Essen Germany; ^13^ Department of Pediatric Hematology/Oncology and BMT University of Wroclaw Wroclaw Poland; ^14^ Pediatric Oncology University of Zürich Zürich Switzerland; ^15^ St. Anna Kinderspital and St. Anna Kinderkrebsforschung e.V. Vienna Austria; ^16^ Department of Women’s and Children’s Health Uppsala University Uppsala Sweden; ^17^ Pediatrics Helsinki University Central Hospital Helsinki Finland; ^18^ Pediatric Surgery University Hospital Tübingen Tübingen Germany; ^19^ Pediatric Hematology and Oncology University Children’s Hospital Münster Münster Germany; ^20^ Department for Children and Adolescents University Hospital, Goethe‐University Frankfurt (Main) Frankfurt Germany; ^21^ Pediatric Hematology and Oncology University of Tübingen Tübingen Germany

**Keywords:** C‐reactive protein, desmoplastic small round cell tumor, maintenance therapy, soft tissue sarcoma, Trousseau’s syndrome

## Abstract

**Background:**

To evaluate optimal therapy and potential risk factors.

**Methods:**

Data of DSRCT patients <40 years treated in prospective CWS trials 1997‐2015 were analyzed.

**Results:**

Median age of 60 patients was 14.5 years. Male:female ratio was 4:1. Tumors were abdominal/retroperitoneal in 56/60 (93%). 6/60 (10%) presented with a localized mass, 16/60 (27%) regionally disseminated nodes, and 38/60 (63%) with extraperitoneal metastases. At diagnosis, 23/60 (38%) patients had effusions, 4/60 (7%) a thrombosis, and 37/54 (69%) elevated CRP. 40/60 (67%) patients underwent tumor resection, 21/60 (35%) macroscopically complete. 37/60 (62%) received chemotherapy according to CEVAIE (ifosfamide, vincristine, actinomycin D, carboplatin, epirubicin, etoposide), 15/60 (25%) VAIA (ifosfamide, vincristine, adriamycin, actinomycin D) and, 5/60 (8%) P6 (cyclophosphamide, doxorubicin, vincristine, ifosfamide, etoposide). Nine received high‐dose chemotherapy, 6 received regional hyperthermia, and 20 received radiotherapy. Among 25 patients achieving complete remission, 18 (72%) received metronomic therapies. Three‐year event‐free (EFS) and overall survival (OS) were 11% (±8 confidence interval [CI] 95%) and 30% (±12 CI 95%), respectively, for all patients and 26.7% (±18.0 CI 95%) and 56.9% (±20.4 CI 95%) for 25 patients achieving remission. Extra‐abdominal site, localized disease, no effusion or ascites only, absence of thrombosis, normal CRP, complete tumor resection, and chemotherapy with VAIA correlated with EFS in univariate analysis. In multivariate analysis, significant factors were no thrombosis and chemotherapy with VAIA. In patients achieving complete remission, metronomic therapy with cyclophosphamide/vinblastine correlated with prolonged time to relapse.

**Conclusion:**

Pleural effusions, venous thrombosis, and CRP elevation were identified as potential risk factors. The VAIA scheme showed best outcome. Maintenance therapy should be investigated further.

## 1 INTRODUCTION

Desmoplastic small round cell tumor (DSRCT) is a rare disease predominantly affecting adolescent and young adult males, which originates and spreads on peritoneal surfaces. Patients usually present with widespread intra‐abdominal metastatic tumors related to serosal surfaces similar to carcinomatosis.^1^ DSRCT consists of small round blue cell nests separated by desmoplastic stroma. It is characterized by the presence of t(11;22)(p13:q12) chromosomal translocation which leads to the fusion of the Ewing sarcoma gene (EWSR1) to the Wilms’ tumor suppressor gene WT1.^2^ First described as a distinct entity in 1989 by Gerald and Rosai,^3^ it remains poorly understood. Despite aggressive multimodal treatment, ~60%‐70% of patients succumb to disease within 2‐3 years.^4^

## 2 PATIENTS AND METHODS

Eligible patients treated from 07/1997 to 06/2015 in the international European trials, CWS‐96,^5^ CWS‐2002P,^6^ and CWS‐SoTiSaR conducted by the Cooperative Weichteilsarkomstudiengruppe CWS, had a confirmed diagnosis in central pathological review, aged <40 years, with no previous malignancy. All CWS trials were prospective and approved by appropriate ethics committees. Written informed consent according to the declaration of Helsinki was obtained from patients or guardians/parents. Data collection was performed as previously described.^6^ Patients received multimodal treatment including surgery, radiotherapy, and chemotherapy, according to the appropriate treatment protocols in use at the time of diagnosis.

### 2.1 Staging and tumor distribution

Disease stage was classified according to IRS and TNM.^7^, ^8^ For purpose of this analysis, localized disease was defined as one single tumor mass; regionally disseminated disease as multiple lesions in one affected cavity, detected by radiological assessment or inspection during surgical intervention. Extraperitoneal metastases were defined as lesions outside the affected cavity or in solid organs.

### 2.2 Thrombosis

Venous thrombosis was diagnosed radiographically at diagnosis before implantation of a central venous line. No systematic screening for thromboses was performed.

### 2.3 Pretreatment blood parameters

Coagulation parameters, blood counts, and C‐reactive protein values (CRP) were evaluated. D‐dimers were graded as normal (<500 µg/L), moderately increased (500‐2000 µg/L), and markedly increased (>2000 µg/L). Scoring for disseminated intravascular coagulation (DIC) was done according to the scoring system of the International Society on Thrombosis and Hemostasis (ISTH).^9^

The inflammatory scores neutrophil‐to‐lymphocyte ratio and platelet‐to‐lymphocyte ratio were calculated by dividing the absolute neutrophil or platelet count, respectively, by the absolute lymphocyte count. The median was chosen as threshold. CRP was considered elevated when >5 mg/L.

### 2.4 First‐line chemotherapy

The induction therapy used was trial‐dependent (Table S1). Early diagnosed patients had received the P6‐scheme according to Kushner.^10^ Regional hyperthermia in combination with platinum‐based chemotherapy^11^ and high‐dose chemotherapy with autologous stem cell rescue were administered on an individual basis.

Hyperthermic intraperitoneal chemotherapy (HIPEC) procedures with cisplatin and doxorubicin in combination with tumor resections were performed, a decision made by the treating oncologist.

### 2.5 Maintenance chemotherapy

Remission was defined as no macroscopic residuals at therapy end (achieved with surgery and/or chemotherapy and/or radiotherapy). Maintenance chemotherapy with oral trofosfamide, idarubicin, etoposide (O‐TIE),^12^ or with cyclophosphamide per os, vinblastine intravenously (Cyc/Vbl)^6^, ^13^, ^14^ was administered (Table S1) trial‐dependent or individually. Some patients received individual therapy with irinotecan/temozolomide or irinotecan/trabectidin. In addition to survival from diagnosis, time to relapse was calculated from end of intensive chemotherapy to eliminate the influence of different lengths of intensive therapies.

### 2.6 Response evaluation

Response was assessed after three chemotherapy courses. For purpose of this analysis, response assessment was based on the CWS evaluation criteria for primary tumors^15^, ^16^: partial response ≥2/3 volume reduction, minor response ≥1/3 but <2/3 reduction, objective response <1/3 reduction, stable disease status idem, progression (≥1/10 volume increase).

In regionally disseminated nodular disease with variable response, the lesion showing the least volume reduction was evaluated.

### 2.7 Local treatment

CWS protocols recommended local treatment of DSRCT analogous to non‐rhabdomyosarcoma (NRSTS). Overall treatment strategies did not change substantially over the years. The mainstay of treatment for NRSTS was primary surgery with preferably complete or wide‐ranging resection after biopsy. In metastatic disease, primary excision or biopsy of the primary tumor and/or metastases was performed and systemic therapy was administered to reduce tumor volume and improve respectability. This was followed by secondary surgery if feasible. Best surgery was defined as the best surgical result obtained at the end of treatment irrespective of procedure numbers. The surgical result was categorized as biopsy only, presence of macroscopic [R2], or microscopic [R1] residual tumor or as resection with free margins [R0].

Radiotherapy was to be administered with a total dose of 32‐54.4 Gy (dose reduced hyperfractionated/accelerated radiotherapy) depending on primary resection status. Postoperative radiotherapy of the primary tumor (and metastases) was recommended. External beam irradiation with 44.8 Gy was recommended if a microscopically complete resection (R0) was not performed. In CWS‐2002P, the recommendation was extended to include patients in whom an R0‐resection had been performed if tumor size was >5 cm or age >10 years. In SoTiSaR, radiotherapy with 50.4 Gy (conventional fractionated irradiation) was added as an alternative. However, individual physician decisions did not always follow these recommendations.

### 2.8 Statistical methods

Statistics were calculated using SPSS^®^ 24 (Armonk, New York, NY, USA). Comparison of distribution was performed with the chi‐square test. Event‐free survival [EFS] and overall survival [OS] were calculated using the Kaplan‐Meier estimator.^17^ For OS, time from diagnosis to death or last follow‐up was calculated, for EFS time from diagnosis to relapse/progression, death, or last follow‐up. Confidence intervals [CI] for the Kaplan‐Meier estimator were computed using Greenwoods Formula^18^ and stated at 95%‐level. For comparison of EFS and OS levels, the log‐rank test was used.

Multivariate analysis was conducted using Cox's proportional hazards regression method to establish independent prognostic significance. A stepwise variable selection procedure (combination of forward and backward selection techniques) was applied to covariates with *P*‐value of at least 0.1 in EFS at univariate analysis. Hazard ratios (HRs) with 95% confidence intervals, calculated according to the Wald method, are reported for significant variables. Statistics were calculated using IBM SPSS^®^ 24 for all other analyses.

## 3 RESULTS

### 3.1 Patient and tumor characteristics

Sixty patients registered from Germany (n = 51), Poland (n = 4), Sweden (n = 2), Austria (n = 2), and Switzerland (n = 1) were eligible. Median age at diagnosis was 14.5 years (6.0‐38.0). Male:female ratio was 4:1 (Table 1). 56/60 patients (93%) had tumors in the abdominal cavity (abdominal and/or retroperitoneal). Four patients had extra‐abdominal tumors, which included four thoracic, one paratesticular, and one parotid gland (all EWSR1‐WT1‐positive). 40/60 (67%) tumors were larger than 10 cm.

**Table 1:** Univariate analysis of characteristics at first diagnosis and pretreatment blood parameters

	60 included patients					25 patients achieving first remission				
	N (%)	3‐y EFS (95% CI)	*P* value	3‐y OS (95% CI)	*P* value	N (%)	3‐y EFS (95% CI)	*P* value	3‐y OS (95% CI)	*P* value
Gender										
Female	11 (18)	27.3 ± 26.3	*0.327*	27.3 ± 26.3	*0.802*	6 (24)	50.0 ± 40.0	*0.599*	50.0 ± 40.0	*0.471*
Male	49 (82)	7.7 ± 7.6		31.9 ± 13.5		19 (76)	19.7 ± 18.6		60.2 ± 23.1	
Age [years]										
≤10	9 (15)	22.2 ± 27.2	*0.201*	64.8 ± 32.3	*0.175*	5 (20)	40.0 ± 42.9	*0.494*	50.0 ± 69.4	*0.225*
10‐21	44 (73)	10.9 ± 9.4		26.7 ± 13.1		18 (72)	26.7 ± 21.0		48.9 ± 23.5	
≥21	7 (12)	0		16.7 ± 29.8		2 (8)	0		50.0 ± 69.4	
Site of primary										
Abdominal	56 (93)	6.7 ± 6.7	***0.01***	27.7 ± 12.2	*0.058*	21 (84)	17.9 ± 17.1	*0.057*	54.0 ± 22.3	*0.31*
Extra‐abdominal	4 (7)	75.0 ± 42.5		75.0 ± 42.5		4 (16)	75.0 ± 42.5		75.0 ± 42.5	
Size of primary										
<10 cm	18 (30)	16.7 ± 17.2	*0.145*	35.4 ± 23.3	*0.59*	7 (28)	42.9 ± 36.7	*0.138*	83.3 ± 29.8	*0.236*
≥10 cm	40 (67)	9.4 ± 9.4		30.3 ± 14.7		18 (72)	20.8 ± 19.4		48.5 ± 23.7	
No information	2 (3)									
Tumor distribution										
Localized^a^	6 (20)	66.7 ± 37.6	***0.004***	83.3 ± 29.8	***0.001***	5 (20)	80.0 ± 35.1	***0.005***	100%	***0.015***
Regionally dissem.	16 (27)	6.3 ± 12.0		33.5 ± 23.9		9 (36)	11.1 ± 20.6		55.6 ± 32.5	
Extrap. metastases	38 (63)	5.3 ± 7.1		22.1 ± 13.3		11 (44)	18.2 ± 22.7		40.4 ± 30.4	
Extraperitoneal metastases in single or multiple organ										
No distant mets	22 (37)	22.7 ± 17.4	*0.134*	47.7 ± 21.4	***0.004***	14 (56)	35.7 ± 25.1	*0.488*	71.4 ± 23.7	***0.038***
Single organ mets	16 (27)	12.5 ± 16.3		27.5 ± 22.5		6 (24)	33.3 ± 37.6		60.0 ± 42.9	
Multiple organ mets	22 (37)	0%		18.2 ± 16.1		5 (20)	0%		20.2 ± 35.1	
Effusion										
No effusion	37 (62)	13.0 ± 11.0	***0.005***	39.9 ± 16.5	***<0.001***	18 (72)	26.7 ± 21.0	*0.347*	57.6 ± 24.1	***0.008***
Ascites	14 (13)	14.3 ± 18.4		26.8 ± 24.1		5 (20)	40.0 ± 42.9		80.0 ± 35.1	
Pleural effusion	3 (5)	0%		0%		2 (8)	0%		0%	
Ascites + pleural eff	6 (10)	0%		0%		0 (0)				
Venous thrombosis										
No	53 (88)	12.6 ± 9.2	***<0.001***	34.7 ± 13.3	***<0.001***	23 (92)	29.0 ± 19.2	***<0.001***	61.8 ± 21.0	***<0.001***
Yes	4 (7)	0%		0%		2 (8)	0%		0%	
No information	3 (5)									
Elevation of CRP										
No	17 (28)	29.4 ± 21.8	***0.002***	51.8 ± 24.3	***0.009***	10 (40)	50.0 ± 31.0	***0.011***	78.8 ± 26.3	*0.052*
Yes	37 (62)	4.1 ± 7.1		25.6 ± 14.9		15 (60)	10.0 ± 17.2		41.3 ± 26.7	
No information	6 (10)									
Thrombocytes > 350/nL										
No	40 (67)	14.6 ± 11.2	*0.318*	32.8 ± 14.9	*0.588*	17 (68)	34.3 ± 23.1	*0.117*	63.3 ± 23.5	*0.229*
Yes	16 (27)	6.3 ± 12.0		34.4 ± 24.1		8 (32)	12.5 ± 22.9		43.8 ± 36.8	
No information	4 (6)									
Neutrophil‐to‐lymphocyte ratio										
<median	24 (40)	15.4 ± 13.9	*0.379*	41.2 ± 20.0	*0.124*	11 (44)	*22.2* ± 25.1	*0.525*	68.6 ± 23.7	*0.826*
>median	26 (43)	11.1 ± 13.3		27.8 ± 18.4		12 (48)	36.4 ± 28.4		56.3 ± 29.0	
No information	10 (17)					2 (8)				
Platelet‐to‐lymphocyte ratio										
<median	27 (45)	18.5 ± 14.7	*0.266*	35.0 ± 18.4	*0.356*	13 (52)	25.0 ± 24.5	*0.715*	51.5 ± 31.0	*0.781*
>median	27 (45)	11.1 ± 11.8		33.6 ± 18.6		12 (48)	28.8 ± 25.7		61.5 ± 26.5	
No information	6 (10)									

*P* values are printed in italics, significant *P* values are bold (*P *< 0.05).

a All with evidence of EWSR1‐WT1 fusion transcript

Six patients had a localized tumor (all EWSR1‐WT1‐positive, 3 extra‐abdominal, 3 abdominal). An additional patient with a single primary in the abdomen also had extraperitoneal metastases (liver, lung, mediastinal lymph nodes).

The tumor was regionally disseminated in 53/60 patients (88%). Thirty‐seven of these 53 (70%) had extraperitoneal metastases (Table S2).

23/60 patients (38%) had effusions, 14 ascites, six ascites in combination with pleural effusions, and three presented with pleural effusion only. Malignant cells were evident in 11/18 examined effusions.

Four patients presented with thrombosis before implantation of a central catheter. Two had a thrombosis in the vena cava, one in the iliac with extension into femoral and popliteal veins while the other had a brachiocephalic and jugular thrombosis.

In 56/60 patients, pretreatment laboratory parameters were available, which included coagulation results in 50 (83%). In 15 (25%), which included only two of the patients with thrombosis, D‐dimers were determined. D‐dimers were normal (n = 2), moderately increased (n = 6), and markedly increased (n = 7); the latter included two patients with thrombosis. Scoring for DIC according to the ISTH^9^ was possible in 12 (20%) patients. All patients scored below 5, defined as nonovert DIC (n = 2 score 0; n = 4 score 2; n = 6 score 3).

Leukocytes ranged from 4.07 to 24.00/nL (median 8.1/nL) in 55/56 patients. Median neutrophil‐to‐lymphocyte and platelet‐to‐lymphocyte ratios were 3.09 (0.56‐9.20) and 170 (50.15‐483.67) in 50 and 54 patients, respectively, with available data, and 2.96 (1.14‐9.20) and 165 (61.63‐435.16) in 23 and 25 patients achieving remission. Platelets ranged from 189 to 566/nL in 56/56 patients. 16/56 (27%) had thrombocytes >350/nL. CRP values were available for 54/56 (90%) and were elevated in 37/54 (69%).^19^

### 3.2 Treatment

37/60 (62%) patients received chemotherapy according to the CEVAIE regimen^12^ (ifosfamide, vincristine, actinomycin D, carboplatin, epirubicin, etoposide), 15/60 (25%) according to VAIA^6^ (ifosfamide, vincristine, adriamycin, actinomycin D) and 5/60 (8%) with P6‐regimen^10^ (cyclophosphamide, doxorubicin, vincristine, ifosfamide, etoposide).

Two patients received alternative therapy (NB95 and Hyper‐PEI, respectively). A single patient did not receive chemotherapy due to severe mental retardation.

Response to chemotherapy ranged from partial response to progression (Table 2). In six patients with regionally disseminated disease, a mixed response with reduction of some and stable or increased volume in other lesions was documented.

**Table 2:** Univariate analysis of conducted first‐line therapies

	60 included patients					25 patients achieving first remission				
	N (%)	3‐y EFS (95% CI)	*P* value	3‐y OS (95% CI)	*P* value	N (%)	3‐y EFS (95% CI)	*P* value	3‐y OS (95% CI)	*P* value
Chemotherapy										
P6	5 (8)	0%	***0.008***	20.0 ± 35.1	***0.043***	4 (16)	0%	***0.001***	25.0 ± 42.5	*0.332*
VAIA	15 (25)	38.9 ± 25.3		53.3 ± 25.3		10 (40)	58.3 ± 31.6		80.0 ± 24.7	
CEVAIE	37 (62)	2.7 ± 5.3		22.2 ± 14.1		10 (40)	10.0 ± 18.6		38.9 ± 33.9	
Other	3 (5)					1 (4)	0%		100%	
Response										
Partial (≥2/3)	17 (28)	5.9 ± 11.2	*0.738*	23.5 ± 20.2	*0.532*	9 (36)	11.1 ± 20.6	*0.685*	44.4 ± 35.5	*0.101*
Minor (≥1/3)	12 (20)	16.7 ± 21.2		38.9 ± 28.8		5 (20)	40.0 ± 42.9		80.0 ± 35.1	
Objective (<1/3)	8 (13)	12.5 ± 22.9		37.5 ± 33.5		3 (12)	33.3 ± 53.3		66.7 ± 53.3	
Stable disease	10 (17)	0%		20.0 ± 24.7		3 (12)	0%		33.3 ± 53.3	
Progression (≥1/10)	5 (8)	20.0 ± 35.1		60.0 ± 42.9		2 (8)	50.0 ± 69.4		100%	
No information	8 (13)					3 (12)				
High‐dose chemotherapy with stem cell transplant										
No	51 (85)	11.4 ± 8.8	*0.613*	32.0 ± 13.1	*0.991*	20 (80)	29.2 ± 20.4	*0.546*	68.1 ± 21.2	*0.051*
Yes	9 (15)	11.1 ± 20.6		19.0 ± 31.4		5 (20)	20.0 ± 35.1		0%	
Hyperthermia in combination with platinum‐based chemotherapy										
No	54 (90)	12.3 ± 9.0	*0.173*	30.2 ± 12.7	*0.403*	24 (96)	27.8 ± 18.6	*0.551*	54.9 ± 21.0	*0.651*
Yes	6 (10)	0%		33.3 ± 37.6		1 (4)	0%		100%	
Best surgery at any time										
R0	5 (8)	40.0 ± 42.9	***0.035***	60.0 ± 42.9	***0.005***	5 (20)	40.0 ± 42.9	*0.709*	60.0 ± 42.9	*0.802*
R1	16 (27)	18.8 ± 19.2		45.8 ± 26.1		14 (56)	21.4 ± 21.6		52.4 ± 28.0	
R2	19 (32)	5.3 ± 10.0		25.1 ± 21.0		4 (16)	25.0 ± 42.5		66.7 ± 53.3	
Biopsy only	19 (32)	5.3 ± 10.0		15.8 ± 16.5		2 (8)	50.0 ± 69.4		50.0 ± 69.4	
No information	1 (2)					1 (4)				
Best surgery at any time in combination with HIPEC										
R0	5 (8)	40.0 ± 42.9	*0.123*	60.0 ± 42.9	***0.021***	5 (20)	40.0 ± 42.9	*0.866*	60.0 ± 42.9	*0.933*
R1	13 (22)	15.4 ± 19.6		35.2 ± 27.2		11 (44)	18.2 ± 22.7		41.6 ± 30.6	
R1 + HIPEC	3 (5)	33.3 ± 53.3		100%		3 (12)	33.3 ± 53.3		100%	
R2	17 (28)	5.9 ± 11.2		25.5 ± 21.6		3 (12)	33.3 ± 53.3		33.3 ± 53.3	
R2 + HIPEC	2 (3)	0%		0%		1 (4)	0%		100%	
Biopsy only	19 (32)	5.3 ± 10.0		15.8 ± 16.5		2 (8)	50.0 ± 69.4		50.0 ± 69.4	
No information	1 (2)					0				
Time of best surgery										
Biopsy only	19 (32)	5.3 ± 10.0	*0.071*	15.8 ± 16.5	***0.008***	2 (8)	50.0 ± 69.4	*0.08*	50.0 ± 69.4	*0.11*
Before Chemotherapy	7 (12)	42.9 ± 36.7		85.7 ± 25.9		5 (20)	60.0 ± 42.9		100%	
Up to 3rd round	12 (20)	8.3 ± 15.7		25.0 ± 24.5		9 (36)	11.1 ± 20.6		33.3 ± 30.8	
After 3rd round	21 (35)	0%		34.2 ± 21.8		9 (36)	0%		57.1 ± 39.0	
Irradiation										
No	40 (67)	5.0 ± 6.7	*0.228*	20.6 ± 12.7	***0.021***	16 (64)	22.2 ± 27.2	*0.591*	44.4 ± 32.5	*0.798*
Yes	20 (33)	25.0 ± 19.0		53.7 ± 22.3		9 (36)	31.3 ± 22.7		67.3 ± 23.5	
Metronomic chemotherapy										
No maint.						6 (24)	0%	*0.056*	33.3 ± 37.6	*0.193*
O‐TIE						11 (44)	24.2 ± 27.0		51.9 ± 30.8	
Cyc/Vbl						5 (20)	60.0 ± 42.9		80.0 ± 35.1	
Irinotecan‐containing						3 (12)	33.3 ± 53.3		100%	
						**N (%)**	**3‐y RFS** **(95% CI)**	***P* value**		
Metronomic chemotherapy evaluated in patients who achieved a first remission										
No maint.						6 (24)	0%	***0.006***		
O‐TIE						11 (44)	12.1 ± 21.8			
Cyc/Vbl						5 (20)	60.0 ± 42.9			
Irinotecan‐containing						3 (12)	66.7 ± 53.3			

RFS, relapse‐free survival calculated from end of intensive therapy.

*P* values are printed in italics, significant *P* values are bold (*P *< 0.05).

Nine patients received high‐dose chemotherapy with autologous stem cell transplantation, and six other patients were treated with regional hyperthermia in combination with platinum‐based chemotherapy^11^ in addition to standard chemotherapy.

21/60 (35%) patients underwent complete resection of primary tumor manifestations (R0 n = 5, R1 n = 16). Two patients with R1 resection suffered disease progression shortly after resection and therefore were not considered as having achieved remission. Five patients were treated with HIPEC, three following R1‐ and two after R2‐resection.

### 3.3 Outcome

Overall, 51/60 patients died, 49 of disease and two of therapy‐related causes (organ failure after chemotherapy, postoperative complications with peritonitis after anus praeter relocation). With a median follow‐up of 3.2 years (1.2‐10.6), 9/60 patients were alive, six in 1st, one in 2nd remission, one lost to follow‐up without ever having achieved remission, and one in first relapse with active disease (Table S4).

**Figure 1:**
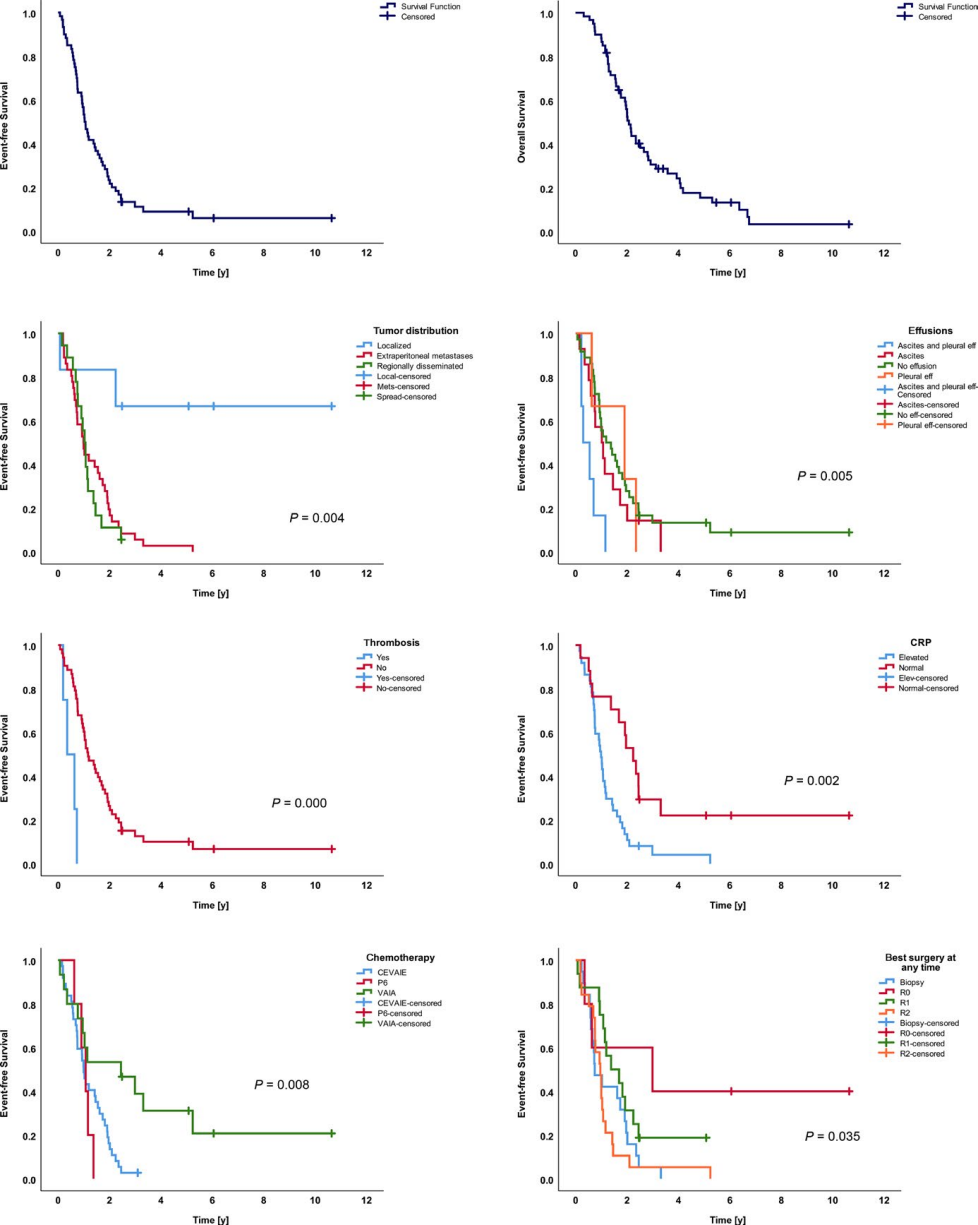
Event‐free and overall survival probability of 60 DSRCT patients. Event‐free survival probability according to the tumor distribution, the existence of effusions, thrombosis, elevated pretreatment CRP‐value, and the conducted first‐line chemotherapeutic regimens and best surgical result at any time in first‐line therapy

Based upon follow‐up data as of March 2017, 3‐year EFS and 3‐year OS were 11% (±8 CI 95%) and 30% (±12 CI 95%; Figure 1).

**Figure 2:**
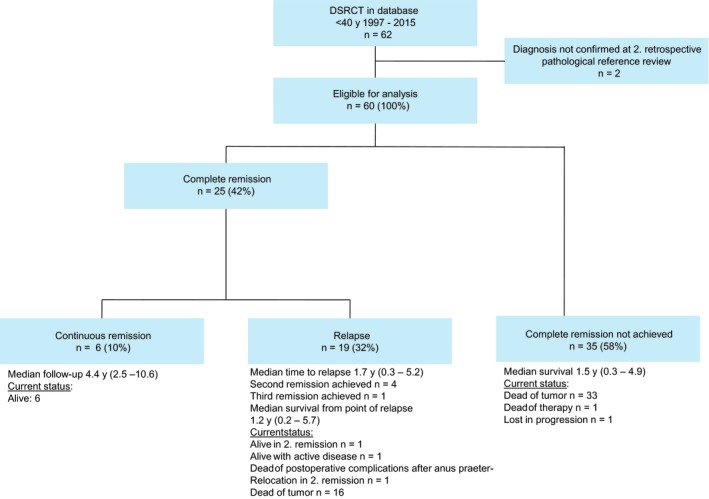
Flow diagram of evaluated patients

25/60 patients (42%) achieved a complete remission at all disease sites, 6/25 (10%) remained in continuous remission and 19/25 (32%) remained in relapsed (Figure 2). For those 25 patients achieving remission, 3‐year EFS and 3‐year OS were 26.7% (±18.0 CI 95%) and 56.9% (±20.4 CI 95%), respectively.

35/60 patients (58%) did not achieve remission and died in a median of 1.5 years.

Among the four patients initially presenting with thrombosis, two showed progression while still on chemotherapy, while the remaining suffered early recurrences. None survived longer than 1.5 years.

### 3.4 Outcome according to surgery

In all those 19 patients who only underwent biopsy as best surgical procedure, an event (disease progression or relapse) was documented in a median of 0.7 years (range 0.2‐3.3). Median overall survival was 1.6 years (0.6‐5.3). None survived. In those other 19 patients with R2‐resection as best surgical result at any time, all had an event in a median of 1.0 years (0.2‐5.2). Median overall survival was 1.7 years (0.3‐6.4). Two were alive at the cutoff date, thereof one lost in progression and the other in first relapse with active disease. Of those 16 patients with R1 resection, 13 had an event in a median of 1.2 years (0.1‐2.4). Median overall survival was 2.6 years (1.1‐6.8). Four patients were in first remission at the cutoff date with a follow‐up of 5.5, 3.2, 2.5, and 2.5 years, respectively. Five were alive. Of those five patients with R0‐resection, 3 had an event in a median of 0.6 years (0.3‐3.0). Median overall survival was 4.2 years (1.0‐10.6). Two were alive at the cutoff date with a follow‐up of 10.6 and 6.1 years, respectively.

### 3.5 Outcome according to chemotherapy

The five patients treated according to the P6‐regimen had a median EFS of 12.9 months (7.6‐16.5), 15 treated with VAIA 29.4 months (1‐127.7), and 37 who received CEVAIE 12.0 months (22.1‐306.7). The remaining patient survived 9 months with palliative care. The VAIA scheme correlated with increased chance of R0 or R0/R1 resection (Table S3).

**Figure 3:**
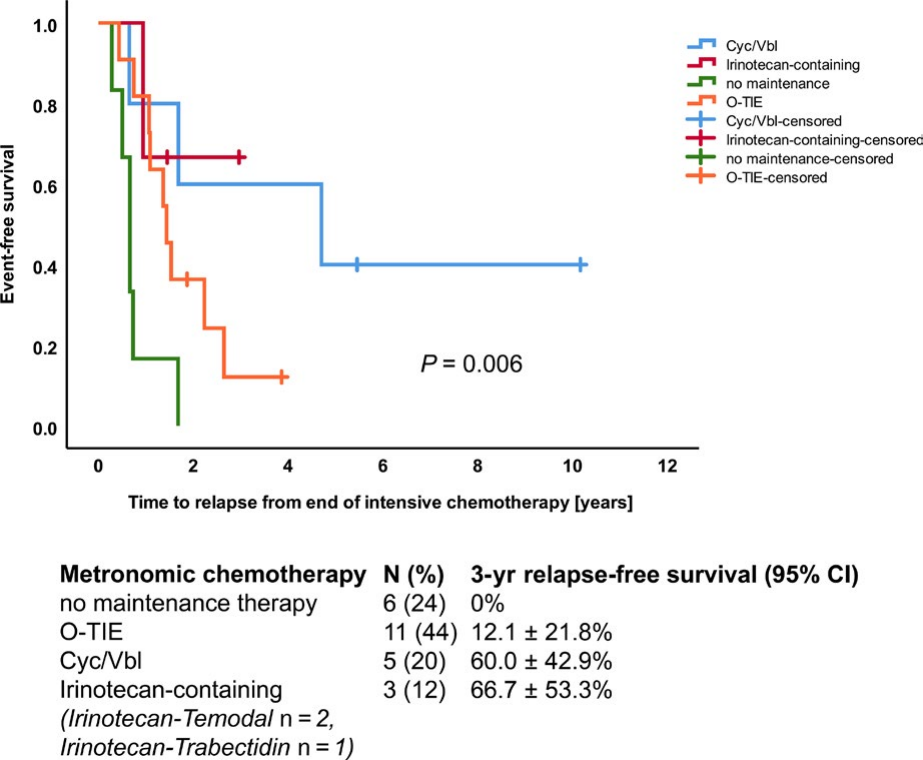
The effect of additional maintenance therapy after the end of intensive chemotherapy evaluated in 25 patients who achieved a first complete remission

Potential correlations between maintenance therapy and outcome were analyzed in the subgroup of 25 patients achieving remission (Table 2, Figure 3). In six patients without maintenance therapy, the median time to relapse calculated from the end of intensive chemotherapy was 8.0 months (3.4‐20.2), in 11 patients with O‐TIE 17.2 months (5.2‐31.8), in five patients with Cyc/Vbl 56.5 months (7.8‐122.0), and in three patients with irinotecan‐containing metronomic therapy 17.4 months (11.3‐35.6).

### 3.6 Pattern of relapse

Among those 19 patients with relapse, five suffered recurrence with merely intraperitoneal lesions. Nine other patients suffered metastatic recurrence with extraperitoneal lesions at different sites (n = 1 lung and bone lesions, n = 1 lymph node supraclavicular, n = 1 bone lesions, n = 1 mediastinal mass and lung lesions, n = 1 lung and liver lesions, n = 1 lung lesions, n = 1 one spleen lesion, n = 1 liver and brain lesions, and n = 1 pleural sarcomatosis). Five patients suffered relapse with combined intra‐ and extraperitoneal lesions. Extraperitoneal lesions were documented at liver and skin (n = 1), paratracheal and bone (n = 1), thoracal and lung (n = 1), thoracic lesions subpleural (n = 1), the site of the affected distant lymph nodes was not documented (n = 1).

### 3.7 Uni‐ and multivariate analyses

In univariate analysis, extra‐abdominal site, localized disease, no effusion or ascites only, absence of thrombosis, and normal CRP were associated with a significantly increased EFS (Table 1). Univariate analysis of metastatic sites did not show any differences in EFS whereas the existence of liver metastases correlated with reduced OS (Table S2).

Among first‐line treatment, chemotherapy with VAIA and complete resection of all primary tumor lesions at any time correlated with prolonged EFS (Table 2).

The distribution of prognostic factors among the chemotherapeutic regimens is shown in Table S3.

A multivariate analysis was conducted to establish independent prognostic significance of the evaluated factors (Table 3). Absence of thrombosis and chemotherapy according to the VAIA regimen remained significant.

**Table 3:** Multivariate analysis of factors predicting increased risk of progression or relapse

Variables	EFS hazard ratio	Confidence interval (95%)	*P*‐value
Site of primary tumor			
Abdominal	1		
Extra‐abdominal	1.958	0.12‐32.13	0.638
Tumor distribution			
Localized	1		
Regionally disseminated	7.837	0.59‐104.90	0.120
Extraperit. Metastases	5.582	0.49‐63.21	0.165
Effusions			
No effusion	1		
Ascites	1.044	0.44‐2.50	0.923
Pleural effusion	0.684	0.17‐2.80	0.597
Ascites + pleural effusion	2.165	0.35‐13.57	0.410
Venous thrombosis			
No	1		
Yes	10.96	2.77‐43.31	**0.001**
Elevation of CRP			
No	1		
Yes	1.231	0.50‐3.00	0.648
Chemotherapy			
VAIA	1		
CEVAIE	3.17	1.13‐8.90	**0.029**
P6	2.58	0.47‐14.26	0.277
Best surgery at any time			
R0	1		
R1	0.75	0.13‐4.19	0.741
R2	1.22	0.24‐6.15	0.807
Biopsy	0.88	0.15‐5.09	0.890

Bold values indicate statistical significance

## 4 DISCUSSION

This study of 60 DSRCT patients confirms the poor prognosis. A small subgroup with reasonable outlook for long‐term, disease‐free survival could be identified, three individuals with localized and extra‐abdominal disease, without pleural effusions, or thrombosis and normal CRP who were subsequently treated by complete tumor resection in conjunction with VAIA chemotherapy. All three patients were disease‐free at last follow‐up (6.1, 5.5, 2.5 years).

An inherent weakness of this study is the small number of patients due to the fact that DSRCT has only been recently described and is a very rare tumor. Nevertheless, to the best of our knowledge, it is the largest series of DSRCT patients enrolled in prospective trials to date. However, our analysis is retrospective and the base‐line blood parameters are nonspecific. Further evaluation should be prospective and standardized.

Characteristics were similar to previous reports.^20^, ^21^ Patients were principally adolescent, male and had large and widespread abdominal and/or retroperitoneal tumors. The majority had extraperitoneal metastases. No typical metastatic pattern was identifiable. No specific risk for disease progression or relapse of any specific metastatic site could be revealed. Nevertheless, though the existence of liver metastases did not correlate with reduced EFS in our series, it correlated with a reduced overall survival probability. Liver metastases have been observed as a potential prognostic factor before.^22^, ^23^ No specific pattern of disease recurrence could be determined; therefore, we were not able to identify specific treatment weaknesses.

In contrast to the literature, existence of extraperitoneal metastases did not correlate with survival.^1^, ^24^, ^25^ This supposed difference might have been caused by the fact that others had not differentiated between localized and regionally disseminated disease. However, others had estimated tumor burden based on peritoneal cancer index but survival did not differ.^24^, ^25^

In contrast to all other solid tumors, size and evidence of distant metastases did not correlate with survival. One cause might be the unique nature of dissemination. It seems that from the point, the cells lose the ability to organize within a single node and start disseminating into multiple nodules, survival decreases significantly. It remains to be elucidated whether this is due to the fact that local therapy is restricted or whether regionally disseminated disease indicates considerably more aggressive biology.

Though the presence of effusions has been described in DSRCT,^26^ prognostic impact is unclear. In our series, pleural effusions correlated with inferior survival.

Four patients aged 8, 10, 11, and 17 years presented with venous thrombosis—detected coincidentally in the initial staging investigations. The association between thrombosis and cancer is well known as the Trousseau's syndrome.^27^ Occurrence varies by age and cancer type.^28^, ^29^, ^30^, ^31^, ^32^ Pathogenesis is multifactorial.^33^ A positive association can be observed with 1‐year mortality of the cancer type as a measure of biological aggressiveness and associated thrombogenic potential.^29^, ^34^ In our series, coagulation activation with thrombus formation seems to indicate more aggressive disease. Unfortunately, detailed analysis of the coagulation cascade was not possible. In only 15/60 (25%) patients, the necessary coagulation values including D‐dimers were determined. Elevation was evident in 13 (86%). It is debatable, whether D‐dimers alone or the ISTH scoring system is sufficient to capture or even quantify the pivotal phenomenon.^35^, ^36^

One contributing factor to coagulation activation is inflammation, which plays an important role in tumorigenesis.^37^ Inflammatory scores are predictive in numerous cancers.^19^, ^38^ In our series, merely CRP correlated with survival. As there are no acknowledged standard values for the applied ratios,^39^ the median was chosen.^40^ However, the predictive significance of CRP suggests need to determine clinical utility. It seems worth mentioning that among those 17 patients with normal CRP, five had localized, four regionally distributed, and eight widespread disease with extraperitoneal metastases.

Of interest are the underlying mechanisms. Malignancy is characterized by destructive tumor cell invasion of vascularized organ tissue and formation of leaky new vessels, so‐called tumor angiogenesis. The procoagulant phenotype is likely to have profound effect on the hemostatic system as result of plasma leakage into the interstitial space, secretion of inflammatory cytokines, and direct invasion of tumor cells into blood or lymphatic vessels.^27^ Coagulation activation, inflammation, and tumor biology form a triangular network with reciprocal interactions.^33^, ^41^ The elucidation of these mechanisms might help to understand DSRCT biology.

The most effective chemotherapeutic regime still is debated. Most combinations are based on alkylating agents, similar to those in other small round cell tumors or Ewing sarcomas, which also carry EWSR1 fusions.^25^, ^42^ Farhat reported five patients with disease stabilization lasting 4‐9 months using a regimen consisting of cisplatin, etoposide, cyclophosphamide, and an anthracycline.^43^ Kushner reported 12 patients with median survival of 19 months with the P6‐protocol.^10^ Bertuzzi reported median survival of 14 months in 17 patients treated in two prospective studies with induction chemotherapy of ifosfamide, epirubicin, and vincristine.^44^, ^45^ Wong reported median time to progression of 14.6 months in 13 patients treated according to the VIDE‐regimen (vincristine, ifosfamide, doxorubicin, etoposide).^20^ Another report states survival of 1.7 years in a single case with VIDE.^46^ The use of other agents has been reported, including irinotecan, temozolomide, and vinorelbine, but none of them showed superiority.^47^, ^48^ Evidence is emerging on trabectedin.^49^, ^50^, ^51^, ^52^, ^53^ Activity was reported for pazopanib^54^, ^55^ and eribulin.^56^ A recent report gave negative results for imatinib.^57^ Limited activity of the antiangiogenics sunitinib, sorafenib, and bevacizumab is reported.^58^, ^59^, ^60^

In our series chemotherapy according to the VAIA scheme correlated with longer EFS than did the other investigated regimes. The median EFS of 15 patients treated with VAIA was 29.4 months. To the best of our knowledge, this is the first assessment of the VAIA scheme in DSRCT. Though the distribution of prognostic factors differed among the three analyzed chemotherapeutic regimens, the independent effect of the VAIA scheme was proven in the Cox regression analysis.

However, the fact that response to chemotherapy did not correlate with outcome is striking. 2/6 (33%) survivors had progressive disease. They were then treated with aggressive surgery.

6/60 (10%) patients showed mixed response of different tumor nodes suggesting heterogenous biology, which should be considered when adopting targeted therapeutic approaches.

In accordance with the recent literature, high‐dose chemotherapy did not correlate with survival.^61^ The value of hyperthermia remains to be specified. However, in our series, patients did not benefit.

While there is considerable investment in the development of innovative therapies, metronomic approaches have been underinvestigated. In our series, metronomic therapy with cyclophosphamide/vinblastine correlated with prolonged time to relapse.

The crucial and decisive role of surgery can be confirmed.^42^, ^62^ In our series, no patient who did not have a R0 resection or R1 resection survived long‐term or was disease‐free at the cutoff date. More precisely, of those 38 patients with only biopsy or R2‐resection, only two were alive at the cutoff date, thereof one lost in progression and the other in first relapse with active disease. However, the feasibility of surgery due to extensively disseminated disease remains a problem. Interestingly, in the subgroup of 25 patients achieving remission, extent of surgery was not predictive of outcome. Two patients who only underwent biopsy achieved remission with chemotherapy alone, one with simultaneous irradiation. Unfortunately, the additional value of HIPEC^1^, ^24^, ^63^, ^64^ cannot be further elucidated as only 5 patients with different resection results received HIPEC.

Although the CWS trials included radiotherapy recommendations, most patients were irradiated following an individual concept due to extensive disease. Radiotherapy mainly consisted of focused irradiation to particular tumor sites or nonresectable nodes. Whole‐abdominal irradiation was performed in two patients. Thus, a reliable conclusion with regard to the specific effect of radiotherapy in DSRCT remains difficult. In our series, irradiation did not correlate with survival. Other authors suggested that a multimodal concept combining chemotherapy, surgery, and irradiation enables prolonges survival.^25^, ^63^, ^65^ In the future, the application and evaluation of standardized radiotherapeutic concepts and/or techniques are necessary to specify its potential benefit.^22^, ^23^

Nevertheless, in our series no specific pattern of disease recurrence was evident, so we were not able to identify specific treatment weaknesses.

In summary, the VAIA scheme could be specified as best chemotherapy in a multivariable model. The effect of maintenance therapy warrants further investigation. Pleural effusions, venous thrombosis, and CRP elevation were identified as novel potential risk factors for adverse outcomes. The elucidation of the underlying coagulation and inflammatory mechanisms may lead to a better understanding of disease biology, especially when investigating the observed coagulatory and inflammatory phenomena in conjunction with genetic analysis.

## CONFLICT OF INTEREST

None of the authors declared a conflict of interest related to the submitted material.
