# Supplementary material for: Desmoplastic small round cell tumors: Multimodality treatment and new risk factors
Source: Cancer Med. 2019 Jan 16;8(2):527–42. doi: 10.1002/cam4.1940 (PMC6382921; doi:10.1002/cam4.1940)
Supplement: Supplementary file 1 [file CAM4-8-527-s001.docx]

Supplementary Table 1. List of chemotherapeutic agents contained in the different schemes

|  | **VAIA** | **CEVAIE** | **O-TIE**  *(8* courses) | **Cyc/ Vbl**  *(7 courses)* |
| --- | --- | --- | --- | --- |
| **Ifosfamide** | 18,000 | 18,000 |  |  |
| **Cyclophosphamide** |  |  |  |  |
| **Doxorubicin** | 160 |  |  |  |
| **Epirubicin** |  | 150 |  |  |
| **Actinomycin-D** | 1.5 | 1.5 |  |  |
| **Vincristine** | 10.5 | 10.5 |  |  |
| **Etoposide** |  | 450 |  |  |
| **Carboplatin** |  | 500 |  |  |
| **Trofosfamide p.o.** |  |  | 12,000 |  |
| **Idarubicin p.o.** |  |  | 80 |  |
| **Etoposide p.o.** |  |  | 2,000 |  |
| **Cyclophosphamide p.o.** |  |  |  | 7,350 |
| **Vinblastine** |  |  |  | 63 |

Cumulative doses of chemotherapeutic agents [mg/m^2^] contained in 3 courses of the respective regime in primary therapy (VAIA, CEVAIE) and in 6 months of the respective maintenance therapy O-TIE and Cyc/Vbl
