# Supplementary material for: Desmoplastic small round cell tumors: Multimodality treatment and new risk factors
Source: Cancer Med. 2019 Jan 16;8(2):527–42. doi: 10.1002/cam4.1940 (PMC6382921; doi:10.1002/cam4.1940)
Supplement: Supplementary file 2 [file CAM4-8-527-s002.docx]

Supplementary Table 2. Univariate analysis of metastatic sites in 60 DSRCT patients

|  | ***N*** | **(%)** | **3yrs EFS**  **(95% CI)** | ***p* value** | **3yrs OS**  **(95% CI)** | ***p* value** |
| --- | --- | --- | --- | --- | --- | --- |
| **Affection of distant lymph nodes**  no  yes | 43  17 | (72)  (28) | 14.0±10.4%  5.9±11.2% | *0.406* | 33.6±14.7%  23.5±20.2% | *0.109* |
| **Liver metastases**  no  yes | 36  24 | (60)  (40) | 19.4±12.9%  0% | *0.198* | 36.5±16.3%  22.4±17.2% | ***0.036*** |
| **Lung metastases**  no  yes | 50  10 | (83)  (17) | 14.0±9.6%  0% | *0.958* | 35.0±13.7%  10.0±18.6% | *0.064* |
| **Pleura metastases**  no  yes | 49  11 | (82)  (18) | 11.4±9.6%  9.1±17.1% | *0.536* | 33.7±13.5%  27.3±26.3% | *0.266* |
| **Bone metastases***  no  yes | 53  7 | (88)  (12) | 12.6±9.2%  0% | *0.265* | 32.8±13.1%  14.3±25.9% | *0.055* |

*thereof 4 with additional bone marrow involvement
