# Supplementary material for: Desmoplastic small round cell tumors: Multimodality treatment and new risk factors
Source: Cancer Med. 2019 Jan 16;8(2):527–42. doi: 10.1002/cam4.1940 (PMC6382921; doi:10.1002/cam4.1940)
Supplement: Supplementary file 3 [file CAM4-8-527-s003.docx]

Supplementary Table 3.

Type of chemotherapy and increased chance of R0 or R0/R1 resection

(excluded from test: patients with primary R0- and R1-resection, “other” chemotherapy and no information on best surgery at any time)

| **Chemotherapy** | **no further resection performed during treatment** | **R2** | **R1** | **R0** | **Chi^2^** | ***p* value** |
| --- | --- | --- | --- | --- | --- | --- |
| P6  VAIA  CEVAIE | 0  1  16 | 1  5  13 | 2  4  6 | 1  2  0 | 14.622 | ***0.02341*** |

| **Chemotherapy** | **no further resection performed during treatment** | **R2** | **R1/ R0** | **Chi^2^** | ***p* value** |
| --- | --- | --- | --- | --- | --- |
| P6  VAIA  CEVAIE | 0  1  16 | 1  5  13 | 3  6  6 | 11.764 | ***0.01919*** |

Prognostic parameters and type of chemotherapy

(* “other chemotherapy” and missing values for the analyzed risk parameters excluded from test)

| **Risk Parameter** | **Total** | **other*** | **P6** | **VAIA** | **CEVAIE** | **Chi^2^** | ***p* value** |
| --- | --- | --- | --- | --- | --- | --- | --- |
| **Gender**  female  male | 11 (18)  49 (82) | 0  3 | 1  4 | 4  11 | 6  31 | 0.7502 | *0.6872* |
| **Age [years]**  ≤10  10-21  ≥21 | 9 (15)  44 (73)  7 (12) | 0  3  0 | 0  4  1 | 2  12  1 | 7  25  5 | 2.0286 | *0.7305* |
| **Site of primary**  abdominal  extra-abdominal | 56 (93)  4 (7) | 3  0 | 4  1 | 12  3 | 37  0 | 7.958 | ***0.0187*** |
| **Size of primary**  <10cm  ≥10cm  no information***** | 18 (30)  40 (67)  2 (3) | 1  2  0 | 2  3 | 3  12 | 12  23 | 1.2163 | *0.5444* |
| **Tumor distribution**  localized  regionally dissem.  extrap. metastases | 6 (20)  16 (27)  38 (63) | 0  1  2 | 0  4  1 | 5  6  4 | 1  5  31 | 26.067 | ***0.0000*** |
| **Effusion**  no effusion  ascites  pleural effusion  ascites + pleural eff | 37 (62)  14 (13)  3 (5)  6 (10) | 3  0  0  1 | 2  1  1  1 | 10  3  0  1 | 22  10  2  3 | 4.326 | *0.6327* |
| **Venous thrombosis**  no  yes  no information***** | 53 (88)  4 (7)  3 (5) | 3  0  0 | 4  1 | 14  1 | 32  2 | 1.2833 | *0.5264* |
| **Elevation of CRP**  no  yes  no information***** | 17 (28)  37 (62)  6 (10) | 2  1  0 | 1  4 | 6  7 | 8  25 | 2.3932 | *0.3022* |
