# Supplementary material for: Desmoplastic small round cell tumors: Multimodality treatment and new risk factors
Source: Cancer Med. 2019 Jan 16;8(2):527–42. doi: 10.1002/cam4.1940 (PMC6382921; doi:10.1002/cam4.1940)
Supplement: Supplementary file 4 [file CAM4-8-527-s004.docx]

| **Patient** | **Site and size of primary** | **Tumor distribution** | **Effusion** | **Venous thrombosis** | **Pretreatment blood parameters** | **Chemo-therapy** | **Response** | **Metronomic chemotherapy** | **High-dose chemotherapy with stem cell transplant** | **Hyperthermia in combination with cisplatin-based chemotherapy** | **Best surgery at any time** | **Irradiation** |
| --- | --- | --- | --- | --- | --- | --- | --- | --- | --- | --- | --- | --- |
| **Male, 9.9 years**  complete remission achieved,  alive in first remission  follow-up 10.6 years | abdominal  >= 10cm | localized  (EWSR1-WT1-positiv) | no | no | CRP normal, available coagulation parameters normal,  d-dimers not analyzed | VAIA | not available due to primary complete resection | Cyc/Vbl  12 weeks | no | no | primary complete resection (R0) | no |
| **Male, 19.6 years**  complete remission achieved,  alive in first remission  follow-up 6.1 years | extraabdominal (paratesticular)  < 3cm | localized  (EWSR1-WT1-positiv) | no | no | CRP normal, available coagulation parameters normal,  d-dimers not analyzed | VAIA | not available due to primary complete resection | Cyc/Vbl  19 weeks | no | no | primary complete resection (R0) | no |
| **Female, 11.9 years**  complete remission achieved,  alive in first remission  follow-up 5.5 years | extraabdominal (parotid gland)  3-5 cm | localized  (EWSR1-WT1-positiv) | no | no | CRP normal, available coagulation parameters normal except of slightly elevated fibrinogen, d-dimers not analyzed | \| VAIA \| \| --- \| | minor response  (≥1/3) | O-TIE  26 weeks | no | no | primary complete resection (R1) | yes |
| **Male, 14.5 years**  complete remission achieved,  alive in first remission, follow-up 3.2 years | abdominal  >= 10cm | locally spread  (EWSR1-WT1-positiv) | ascites with evidence of tumor cells | no | CRP elevated, available coagulation parameters including d-dimers normal | CEVAIE | progression | second line-chemotherapy with irinotecan/ temodal | no | no | R1-resection in combination with HIPEC after 3 cycles of chemotherapy | yes |
| **Male, 16.8 years**  complete remission achieved,  alive in first remission, follow-up 3.1years | abdominal  >5 - <10cm | locally spread  (EWSR1-WT1-positiv) | ascites with evidence of tumor cells | no | CRP elevated,  available coagulation parameters normal,  d-dimers moderately increased | CEVAIE | partial response (>2/3) | O-TIE  28 weeks | no | no | R1-resection in combination with HIPEC after 8 cycles of chemotherapy | yes |
| **Female, 6.3 years**   \| complete remission achieved, alive in first remission, follow-up 2.5 years \| \| --- \| | extraabdominal (thoracic)  >= 10 cm | localized  (EWSR1-WT1-positiv) | no | no | CRP normal, available coagulation parameters normal, d-dimers moderately increased | \| VAIA \| \| --- \| | \| minor response  (≥1/3) \|  \| \| --- \| --- \| | Irinotecan/ Trabectidin  22 weeks | yes | no | R1-resection after 9 cycles of chemotherapy | yes |
| **Male, 6.6 years**  alive in first relapse with active disease, follow-up 1.7years | abdominal  >5 - <10cm | locally spread with single organ metastases, liver  (EWSR1-WT1-positiv) | no | no | CRP elevated, available coagulation parameters normal except of slightly elevated fibrinogen, d-dimers moderately increased | CEVAIE | minor response  (≥1/3) | no | no | no | R2-resection (tumor reduction >90%) after 7 cycles of chemotherapy in combination with HIPEC | yes |
| **Male, 8.9 years**  Alive in 2^nd^ remission,  follow-up 3.4years | abdominal  >5 - <10cm | localized  (EWSR1-WT1-positiv) | no | no | CRP normal, no coagulation parameters available | CEVAIE | partial response (>2/3) | O-TIE  22 weeks | no | no | primary R1-resection | yes |
| **Male, 36.4 years**  Lost to follow-up without having ever achieved first remission, follow-up 1.2years | abdominal  no information about size | locally spread | no | no | CRP elevated, available coagulation parameters normal | CEVAIE | no information | did not achieve complete remission | yes | no | primary resection (R2) | no |
